# Network based transcription factor analysis of regenerating axolotl limbs

**DOI:** 10.1186/1471-2105-12-80

**Published:** 2011-03-18

**Authors:** Deepali Jhamb, Nandini Rao, Derek J Milner, Fengyu Song, Jo Ann Cameron, David L Stocum, Mathew J Palakal

**Affiliations:** 1 School of Informatics, Indiana University-Purdue University Indianapolis, Indianapolis, IN, 46202, USA; 2 Department of Biology, Indiana University-Purdue University Indianapolis, Indianapolis, IN, 46202, USA; 3 Department of Cell and Developmental Biology, and Regeneration Biology and Tissue Engineering Theme, Institute for Genomic Biology, University of Illinois-Urbana Champaign, Urbana, IL, 61801, USA; 4 Department of Oral Biology, School of Dentistry, Indiana University-Purdue University Indianapolis, Indianapolis, IN, 46202, USA; 5 Center for Regenerative Biology and Medicine, Indiana University-Purdue University Indianapolis, Indianapolis, IN, 46202, USA; 6 American University of Antigua, College of Medicine, University Park, Jabberwock, Coolidge, Antigua, P. O. Box W-1451, West Indies

## Abstract

**Background:**

Studies on amphibian limb regeneration began in the early 1700's but we still do not completely understand the cellular and molecular events of this unique process. Understanding a complex biological process such as limb regeneration is more complicated than the knowledge of the individual genes or proteins involved. Here we followed a systems biology approach in an effort to construct the networks and pathways of protein interactions involved in formation of the accumulation blastema in regenerating axolotl limbs.

**Results:**

We used the human orthologs of proteins previously identified by our research team as bait to identify the transcription factor (TF) pathways and networks that regulate blastema formation in amputated axolotl limbs. The five most connected factors, c-Myc, SP1, HNF4A, ESR1 and p53 regulate ~50% of the proteins in our data. Among these, c-Myc and SP1 regulate 36.2% of the proteins. c-Myc was the most highly connected TF (71 targets). Network analysis showed that TGF-β1 and fibronectin (FN) lead to the activation of these TFs. We found that other TFs known to be involved in epigenetic reprogramming, such as Klf4, Oct4, and Lin28 are also connected to c-Myc and SP1.

**Conclusions:**

Our study provides a systems biology approach to how different molecular entities inter-connect with each other during the formation of an accumulation blastema in regenerating axolotl limbs. This approach provides an in silico methodology to identify proteins that are not detected by experimental methods such as proteomics but are potentially important to blastema formation. We found that the TFs, c-Myc and SP1 and their target genes could potentially play a central role in limb regeneration. Systems biology has the potential to map out numerous other pathways that are crucial to blastema formation in regeneration-competent limbs, to compare these to the pathways that characterize regeneration-deficient limbs and finally, to identify stem cell markers in regeneration.

## Background

Current thinking in regenerative medicine envisions the derivation, from autogeneic somatic cells, of pluripotent cells that can be directed to differentiate into transplantable replacements for cells destroyed by injury or disease [1]. Beyond this, however, is another goal: the chemical induction of regeneration directly at the site of tissue damage [2]. Achievement of this goal will require a deep understanding of the molecular components, networks and pathways that characterize regenerative competence. Urodele amphibians (axolotls, salamanders and newts), which regenerate amputated limbs perfectly throughout larval and adult life, provide a research model that lends itself well to furthering our understanding of this process. Two hundred fifty years after Lazzaro Spallanzani first demonstrated the regeneration of amputated newt limbs [3], we still do not fully understand the mechanisms of this process. Urodele limbs initiate regeneration through the formation of a blastema: a limb bud-like structure composed of undifferentiated progenitor cells. Blastema cells originate by a reverse developmental process in which the tissue matrix near the amputation plane is degraded by proteases, releasing both mature cells that are reprogrammed to a mesenchymal stem cell-like state, and muscle stem cells (satellite cells) [4-7]. Within a few days after amputation, these cells accumulate under the apical epidermal cap (AEC), where they proliferate and are patterned into the missing limb parts.

The ability to form a blastema is what distinguishes urodele limbs from the limbs of most other tetrapod vertebrates that do not regenerate or which regenerate poorly. Thus, understanding the mechanisms that lead to blastema formation is crucial to understanding why urodele limbs regenerate, and why the limbs of other species do not. In general, the reductionist approach has been to study the individual genes or proteins involved in biological processes. With the development of high throughput technology over the last decade, there has been a shift in this approach. The ability to obtain large scale omics data has led to the development of discovery approaches that interrelate the elements of biological processes, revealing networks and pathways of organization in a system [8]. Very few studies so far have analyzed global gene or protein expression patterns during limb regeneration. In the axolotl *Ambystoma mexicanum*, expressed sequence tag (EST) resources have been developed [9] and transcription profiles of denervated vs. innervated limbs have been analyzed [10]. Some global studies have been performed in an anuran amphibian, the frog *Xenopus laevis*. However, unlike urodeles, Xenopus possesses the ability to regenerate lost limbs in early tadpole stages of development, but gradually loses the capability for regeneration as development proceeds, until it is lost completely in adults [11]. Xenopus studies have focused on subtractive hybridization [12]; microarray analysis [13] and proteomics [14] for molecular screening of limb regeneration.

Although extensive research has been carried out to understand how the blastema is formed and which molecular entities are crucial to regeneration, very little is known about the interactive pathways and networks that lead to blastema formation in an amputated limb. Recently, we conducted a proteomic analysis of blastema formation in the amputated limbs of *Ambystoma mexicanum*. Our analysis revealed a number of significant changes in protein expression related to cell signaling, transcription, metabolism, cell protection, and cell cycle [15]. We are now engaged in a broad, systems level analysis of high throughput datasets in limb regeneration. Here we focus on the networks and pathways regulated by the transcription factors (TFs) c-Myc (myc proto-oncogene protein) and SP1 (specificity factor 1), which we found to be connected to 36.2% of the proteins expressed during axolotl limb regeneration blastema formation. In particular, we found that TGF-β1 (transforming growth factor - beta 1) could potentially lead to the activation of SP1 and then to the expression of FN (fibronectin), which is produced by the blastema cells and the AEC. In turn, FN activates c-Myc via integrins and the Wnt pathway. Within these pathways we identified several TFs such as SMAD3 (mothers against decapentaplegic homolog 3), which may be involved in limb regeneration. In addition, Klf4 (kruppel-like factor 4), Oct4 (octamer-binding protein 4), and Lin28, TFs common to embryonic stem cells, induced pluripotent cells (iPSCs) and blastema cells [15-18], were also found to be connected to c-Myc and SP1. Our results highlight the utility of systems biology for understanding complex processes such as limb regeneration.

## Methods

### Processing of Axolotl Proteomics Data

Network and pathway analysis was based on proteomics data obtained at 1, 4 and 7 days post-amputation (dpa) in our study of blastema formation in the hindlimb of the wild-type axolotl, *Ambystoma mexicanum *[15]. In that study, 309 high confidence peptides were identified with significant fold changes relative to control on one or more dpa. Since bioinformatics resources for the axolotl are limited, we identified human orthologs for these peptides. The search was carried out against the *Homo sapiens *database (taxid: 9606) using the BLASTp tool (Basic Local Alignment Search Tool for proteins) available from NCBI (National Center for Biotechnology Information) [19]. The peptides in the original study were five to thirty three amino acids long and were aligned throughout their length for ortholog identification. A human ortholog was used only if the percentage identity between an axolotl: human peptide was greater than 85%; unmatched peptides were excluded from the analysis. Although proteins with lower percentage identities could well be important, LC/MS/MS analysis relies on peptide sequences for alignment, not complete protein sequences, making it essential to set a high percentage identity threshold for stringency. The UniProt database [20] was used to assign the gene name to each of the human orthologs. The Database for Annotation, Visualization and Integrated Discovery (DAVID) [21,22] was used for the assessment of biological processes. The ortholog data was divided into 6 groups with respect to up- and down-regulated proteins at each time point: 1d-, 1d+, 4d-, 4d+, 7d-, and 7d+. Thus, 1d- refers to all down-regulated proteins at 1 dpa, and 1d+ refers to all up-regulated proteins at 1dpa; all other groups are interpreted in a similar manner.

### Network Analysis

#### 1. TF connectivity map

All the human orthologs identified from the axolotl proteomics data were used as a bait to identify TFs connected to these orthologs. Few proteins had upstream (incoming) interactions with these TFs, so only downstream (outgoing) interactions were used to construct a unidirectional connectivity map. Transcription factor identification was done using the Transcription Regulation algorithm from the commercial software MetaCore™ version 5.4, build 19940 (GeneGo, Inc) which is based on manual curation. This algorithm generated sub-networks centered on TFs that have direct links to our bait list data. Transcription factors were ranked according to their p-value, based on hypergeometric distribution [23]. The ranking represents the probability of picking up a TF by chance, considering the number of bait list proteins it mapped to from our data versus the number of genes in the network within the full set of all proteins in the networks. That is, the higher the number of direct interactions for a TF in the given proteomic dataset, the lower is the p-value. A TF connectivity map was constructed using the radial tree layout in Cytoscape [24,25].

#### 2. Upstream receptor identification

Networks were built to specifically target the upstream pathways that activate the TFs c-Myc and SP1. Receptors of upstream pathways were identified using the Analyze Network (Receptor) algorithm from MetaCore™. This algorithm generates a network for each receptor in the input data consisting of the shortest paths from it to the nearest TF. A similar p-value score, as described above, was used for the statistical evaluation of networks.

### Pathway Analysis

The target proteins of c-Myc and SP1 in the bait list as well as the rest of the proteins were evaluated for significant pathways with respect to up and downregulated groups at each time point. Statistical significance was measured by the number of proteins that map onto a given pathway. Hence, this method did not identify pathways for each individual protein, but rather those which are more likely to be prevalent in the groups mentioned above. The significance was calculated on the basis of z-score (a built-in feature of MetaCore™).

## Results

### 1. Temporal and Functional Data Analysis

**Figure 1:**
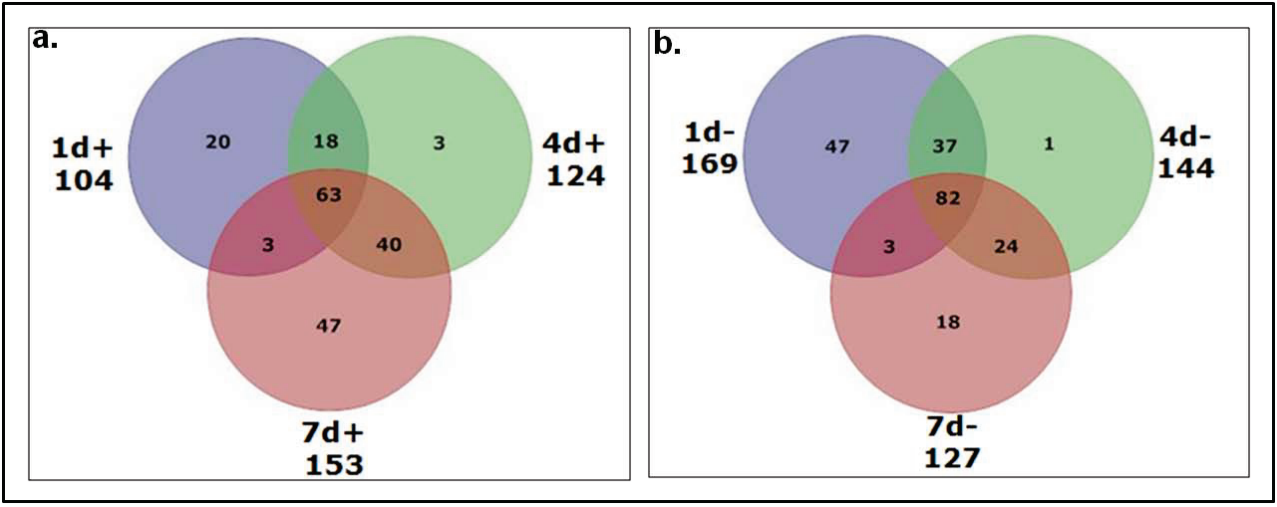
**Venn diagram for a) upregulated and b) downregulated groups**. The diagram shows the number of up and down regulated proteins in the axolotl proteomics data at 1, 4 and 7 day post amputation. 1d+ refers to the upregulated proteins and 1d- refers to the downregulated proteins at day 1. Other time points can be similarly interpreted. The value under each time point shows the total number of proteins up/down regulated at that time point.

Figure 1 depicts Venn diagrams for the proteins up regulated at 1, 4 and 7 dpa (Figure 1a), and down regulated at 1, 4 and 7 dpa (Figure 1b). Figure 1a shows that 7d+ has the highest number of differentially regulated proteins (153) followed by 4d+ (124) and 1d+ (104). Interestingly, the 4d+ group had few unique proteins (3) with most of the proteins (63) up regulated at all three time points, similar to the down regulated group. A functional enrichment analysis by the DAVID tool showed that 20 proteins unique to the 1d+ group were enriched in cell cycle processes. Forty-seven proteins unique to the 7d+ group were enriched in cell structure and motility, and RNA processing. Forty proteins common to the 4d+ and 7d+ groups showed enrichment in metabolism, cell cycle, and mRNA-related processes. Proteins common to all time points were enriched in intracellular protein trafficking, endocytosis, chromatin packaging and neurotransmitter release (possibly in regenerating nerves). Figure 1b shows that 1d- has the maximum number of statistically significant differentially regulated proteins (169) followed by 4d- (144) and 7d-(127). Of these, the majority of proteins (82) were downregulated at all three time points. Only 1 protein was unique to the 4d- group and very few proteins (3) were common to the 1d- and 7d- groups. The functional enrichment analysis revealed that 47 proteins unique to 1d-, 18 proteins unique to 7d-, and 24 proteins common to 4d- and 7d-groups were enriched in cell cycle related processes but more proteins were involved than in the 1d+ group. The 82 proteins common to all time points were enriched in biological processes related to general development, cell structure and motility, muscle carbohydrate metabolism, cell cycle, and mRNA splicing (categories specified by the DAVID tool). A comprehensive list of all 301 proteins from the axolotl proteomics data with their human orthologs, respective gene names and fold change difference is provided in the supplementary material [Additional file 1]. A list of enriched biological processes (obtained using DAVID) for each sector in the Venn diagram can also be found in the supplementary material [Additional file 2].

### 2. Transcription Factor Analysis

To understand a complex biological process such as limb regeneration, it is crucial to elucidate and understand its regulatory machinery. One of the limitations of the LC/MS/MS method used in our original proteomic analysis of blastema formation in axolotl limbs [15] is that it often fails to identify proteins expressed in low amounts and post translationally modified proteins (PTMs) [26,27]. As a result, certain growth factors, signaling molecules and TFs have a higher probability of going undetected. We used the human ortholog bait list to fish out the missed TFs by using the manually curated MetaCore™ database. Significance is based on the number of proteins to which the TFs connected in our data. Supplementary material contains information about the five most significant TFs in each data group (1d- to 7d+), the number of target proteins to which each TF linked from our bait list, a p-value to describe their significance, and the enriched GO processes [Additional file 3].

**Figure 2:**
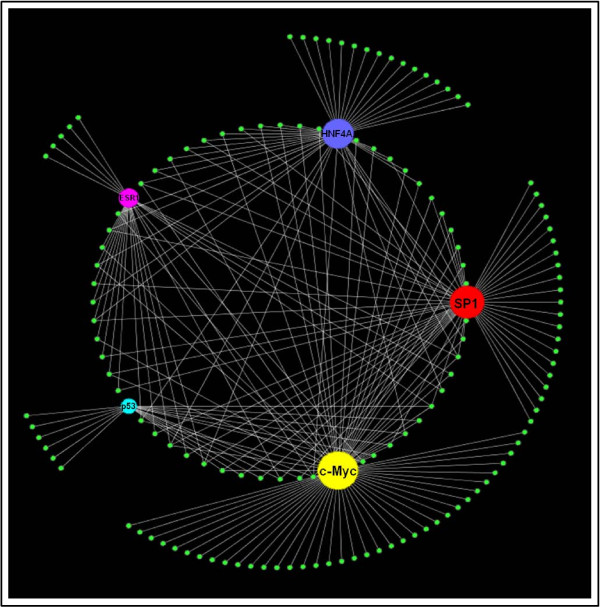
**Transcription factor connectivity in axolotl proteomics data**. The five most highly connected TFs (c-Myc, SP1, HNF4A, ESR1 and p53) and their downstream targets from the bait list proteins (represented by small green circles) are shown here. The size of the TF circle corresponds to its connectivity; a bigger circle entails higher overall connectivity. The targets in the outer circle are unique targets of each TF while those in the inner circle are shared by two or more TFs.

**Figure 3:**
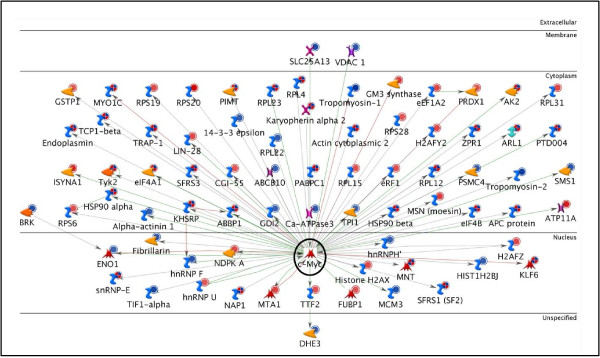
**c-Myc Network**. The network shows c-Myc (enclosed by a black circle) with its 71 targets from the bait list. The horizontal lines separate the proteins in the network into the following categories: extracellular, membrane, cytoplasm, nucleus, and unspecified. The various symbols used in the network have been described in detail in the supplementary information [Additional file 7].

**Figure 4:**
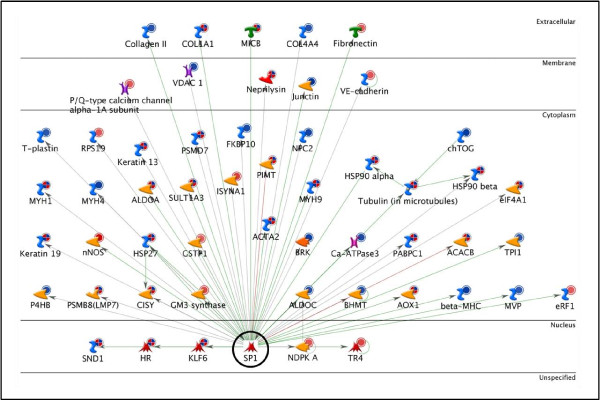
**SP1 Network**. The network shows SP1 (enclosed by a black circle) with its 56 targets from the bait list.

Figure 2 represents the overall connectivity for the five most highly connected TFs from the bait list: (c-Myc, SP1, HNF4A (hepatocyte nuclear factor 4-alpha), ESR1 (estrogen receptor1), and cellular tumor antigen p53). The results show that one hundred thirty-nine (46.2% of the total) proteins in our bait list could potentially be regulated by these five TFs (all the overlapping targets between different TFs were removed to calculate this number). c-Myc was found to have the highest connectivity (71 targets) as well as the maximum number of unique targets. SP1 was the second highest connected TF (56 targets) and also had more unique targets than the other three TFs (excluding c-Myc). Figure 2 also shows that for most of the other three TFs (HNF4A, ESR1, and p53) there was a high degree of overlap (fewer unique targets) since most of their targets were shared by c-Myc and SP1. For this reason, we focused on c-Myc and SP1 in this study. Supplementary table lists the details of all target proteins in the bait list for each TF [Additional file 4].

c-Myc and SP1 were found to regulate 109 unique target proteins from our data. This number was calculated by removing all overlapping proteins between c-Myc and SP1. Thus, c-Myc and SP1 alone were responsible for potentially regulating 36.2% of the target proteins from the bait list. Figures 3 and 4 show the c-Myc and SP1 networks, respectively, with all the target proteins to which they connected.

### 3. Network Construction and Pathway Analysis

Once c-Myc and SP1 were identified as the most significant TFs, further investigation of the interacting upstream and downstream elements for these TFs was carried out. Downstream elements were the target proteins for these TFs in our data (Figures 3 and 4). These networks were statistically found to be the most significant in our data. Many other proteins not identified by our proteomics screen but well established in limb regeneration, such as MMPs (matrix metalloproteinases) [28,29] and annexins [14,15] were also present in these networks. This further validates the significance of the networks with respect to limb regeneration.

**Figure 5:**
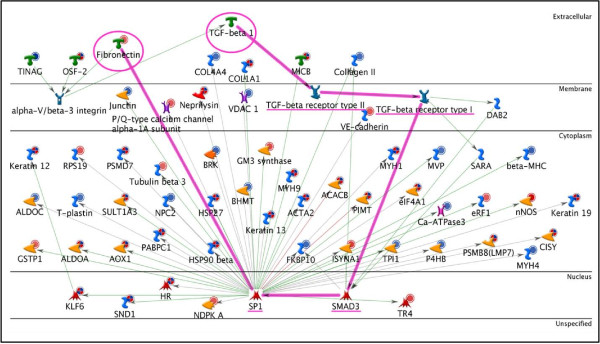
**TGF-β1, SP1, FN network**. The path from TGF-β1 to SP1 that ultimately leads to the activation of FN has been highlighted. The start point (TGF-β1) and end -point (FN) are highlighted with a circle and the connecting proteins are underlined.

**Figure 6:**
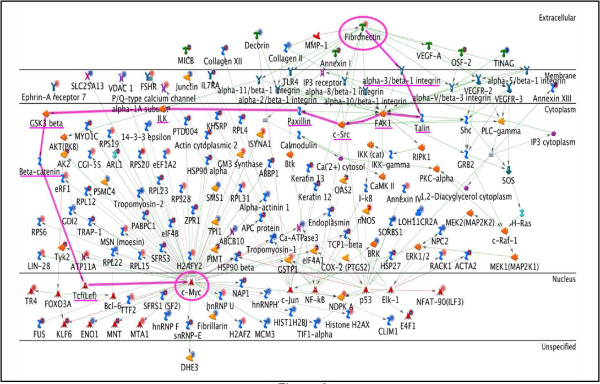
**Fibronectin - c-Myc network**. This network shows that multiple signaling pathways from FN can lead to the activation of c-Myc. The pathway highlighted in pink includes proteins involved in canonical Wnt signaling (GSK3beta, beta-catenin, and Tcf(Lef)).

We identified several canonical pathways known to be present in humans or mice [30-32] spread across these networks. We found that TGF-β1 activates SP1 through SMAD proteins. One of the downstream targets of SP1 is FN (this also is a well established canonical pathway). Fibronectin then activates c-Myc via integrins and the Wnt pathway. These paths are highlighted in Figures 5 and 6.

The most significant pathways regulated by target proteins of c-Myc and SP1 in our data are provided in the supplementary information [Additional file 5]. Supplementary information also provides the pathways that were identified using the remainder of the proteins not regulated by either c-Myc or SP1 [Additional file 6]. Among the pathways identified, several are already well established in limb regeneration such as cytoskeleton remodeling, cell adhesion and development related, thus validating the approach. Some pathways that have been of interest recently in limb regeneration such as cell cycle, immune response, and metabolism were also identified [15].

### 4. Stemness in Limb Regeneration

**Figure 7:**
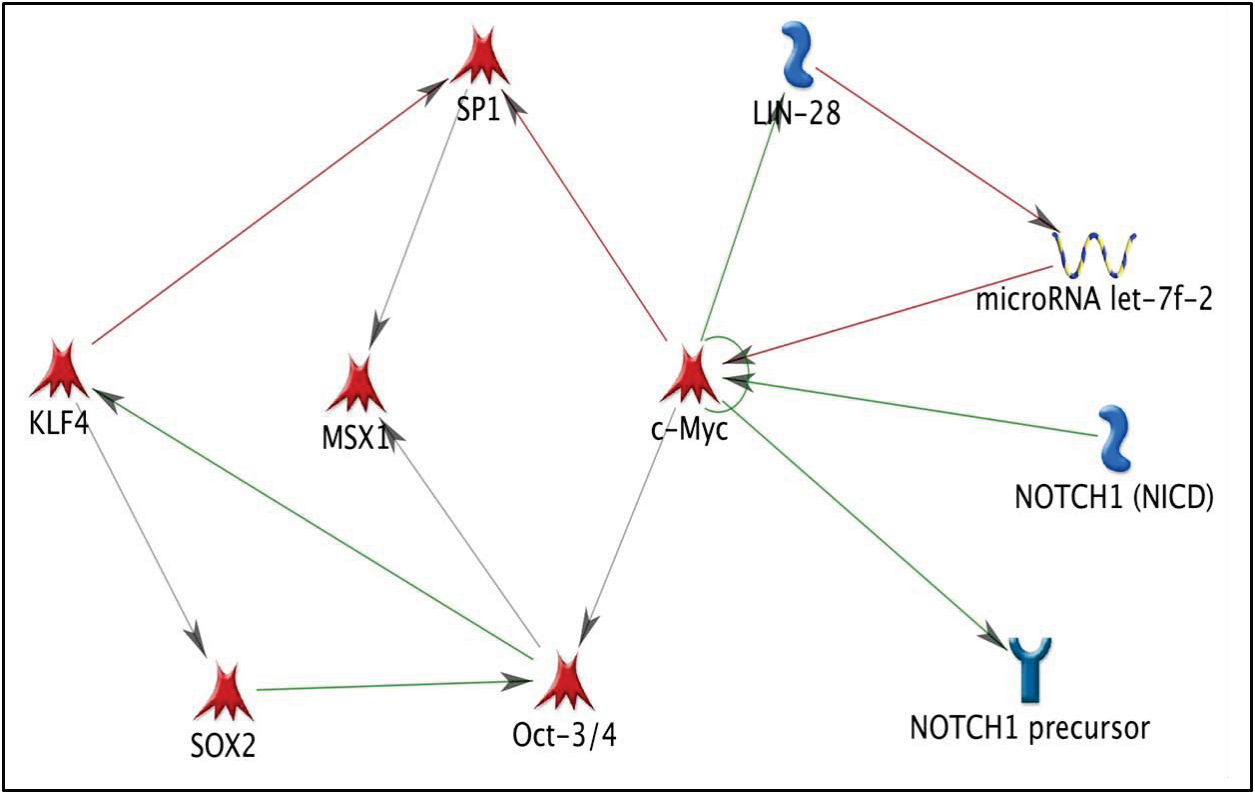
**Stemness in limb regeneration**. This network highlights the relationship of known stem cell markers (Oct3/4, Sox2, KLF4, Lin28) with TFs identified by this study (c-Myc and SP1) and previously identified TFs in limb regeneration (Msx1 and Notch1).

Blastema cells express TFs associated with stemness (e.g., Msx-1) [1,4]. Recently, combinations of the TFs c-Myc, Oct4, Sox2, Klf4, Lin28, and Nanog have been shown to reprogram adult fibroblasts to iPSCs [16,17]. c-Myc has been shown to enhance the ability of Oct4, Sox2 and Klf4 to induce pluripotency up to 10 fold [16]. However, high levels of c-Myc are only transiently required and sustained levels were found to lead to tumors [33,34]. c-Myc, Klf4 and Sox2 have been shown to be expressed in regenerating newt limb tissue, and Lin28 in regenerating axolotl limb tissue [15,18,35,36]. Hence, we constructed a network (Figure 7) that included all of these TFs to evaluate their significance for stemness in a mammalian system. This figure shows how mammalian stem cells might be related to urodele blastema cells. The various symbols used in the network have been described in detail in the supplementary information [Additional file 7].

## Discussion

### 1. Human Ortholog Identification

Our previous proteomics study [15] identified 309 differentially regulated proteins at three time points (1, 4, and 7 dpa) during blastema formation in regenerating axolotl limbs. Here we used the human orthologs of these 309 proteins as bait to identify potential associations with other proteins that were not identified in the proteomics screen. We obtained 301 significant human orthologs for 309 axolotl proteins and used them to construct interactive protein networks. Our focus was on the identification of significant TFs and molecules regulating or regulated by these TFs that might be key to axolotl limb regeneration.

### 2. Temporal And Functional Data Analysis

We first separated the orthologs into up and down regulated groups for each day post-amputation. In both the up and down regulated groups, very few proteins (3 and 1 respectively) were unique to day 4. This suggests that day 4 proteins are involved in carrying out biological processes similar to either day 1 or day 7. Most of the proteins were either up regulated at all time points or down regulated at all time points. Those down regulated at all time points were enriched for proteins involved in development, cell structure and motility, muscle contractile activity, carbohydrate metabolism, cell cycle, and mRNA splicing, whereas those up regulated at all time points were enriched for intracellular protein trafficking, endocytosis, chromatin packaging and neurotransmitter release (possibly in regenerating nerves).

### 3. Transcription Factor Network Analysis

Next, we derived a TF-centric protein interaction network using commercially available MetaCore™ software to identify all human TFs that were connected to at least five proteins in the data. The five most highly connected TFs, c-Myc, SP1, HNF4A, ESR1 and p53, were found to regulate almost half of the proteins (46.2%) in our data. Of these, c-Myc and SP1 alone were found to regulate 36.2% of the proteins. c-Myc was the most highly connected TF (71 target proteins) and also had the highest number of unique targets (not regulated by other TFs) while SP1 had the next highest TFs. For these reasons, we focused on these two TFs.

c-Myc is involved in various biological processes such as proliferation, growth, apoptosis, energy metabolism and differentiation [33,34]. It has been shown to act with β-catenin to inhibit wound healing by interfering with differentiation in chronic ulcers [37] and is expressed in regenerating limb and lens of the newt *Notophthalmus viridescens*. In the regenerating newt limb, *in-situ *hybridization has shown that c-Myc is localized in both the epidermis and subjacent blastema cells. This expression has been correlated with the maintenance of blastema cell proliferation [35,36]. Recently, along with other stem cell factors, c-Myc expression in the regenerating newt limb was found to be highest during the dedifferentiation phase of blastema formation. Expression then decreased at later stages but still remained higher than the control tissue [18]. These studies have related c-Myc to proliferation as well as stemness, but the downstream targets of c-Myc which result in these effects have not been identified. Here, we have identified 71 downstream targets of c-Myc in our bait list. These targets are involved in various biological processes/pathways related to limb regeneration. Using this information, hypotheses about the specific role of c-Myc in limb regeneration can be proposed and tested.

Specificity factor1 was the second highest connected TF (56 target proteins). SP1 is a ubiquitously expressed protein and has varied roles in cell growth, differentiation, apoptosis, angiogenesis, tumorigenesis and immune response. It is known to interact with cyclins which promote the G1/S phase transition, as well as with cyclin-dependent inhibitors that inhibit progression through the cell cycle. Similarly, its target genes include both pro- and anti-apoptotic genes and pro- and anti-angiogenic genes. Specificity factor1 is also linked to chromatin remodeling through its interaction with p300 and histone deacetylases (HDACs) and is known to interact with several TFs including c-Myc in order to activate several downstream target genes. However, SP1 action is highly dependent on its interaction with other members of the SP family and extracellular signals [38-40]. This is the first time SP1 has been identified in relation to limb regeneration and no studies have yet been done on the specific role it plays in limb regeneration.

### 4. Stem Cell Factors In Limb Regeneration

A number of TFs associated with stemness are expressed during blastema formation. Some of these, such as the Hox and Meis genes, regulate pattern formation in the growing blastema [41-46], whereas others such as msx-1, nrad, Klf4, Oct4, Sox2, and Lin28 are associated with dedifferentiation and proliferation [15,18,47-51]. With the exception of Lin28, we did not identify any of these TFs in our proteomic analysis of blastema formation in the axolotl hind limb [15], but Figure 5 demonstrates that they interact with c-Myc and SP1. This suggests that c- Myc and SP1 are central to a network of TFs that regulate mesenchymal stem cell properties of the blastema and that play a role in the nuclear reprogramming of differentiated limb cells to blastema cells.

### 5. Pathway Analysis

Next we mapped out the pathways of two molecules, TGF-β1 and FN, that connect SP1 and c-Myc, respectively, and which are known to be important in mammalian tissue repair and urodele limb regeneration. Within the TGF superfamily, the TGF-β and BMP subfamilies of growth factors are major players in skin wound repair and bone regeneration. Transforming growth factor beta isoforms attract immune cells into skin wounds, and induce the migration and proliferation of skin fibroblasts to form granulation tissue. Transforming growth factor β activates many target genes in wound healing, including connective tissue growth factor (CTGF) and FN [52].

The wound epidermis covering the amputation surface plays a crucial role in blastema formation [11]. Establishment of the wound epidermis after amputation of the Xenopus tadpole tail requires TGF-β signaling [53]. TGF-β and SMAD 2 are up regulated early in formation of the wound epidermis and later in the tissues of the blastema. Inhibition of TGF-β signaling with the inhibitor of SMAD phosphorylation SB-431542 immediately after amputation prevents establishment of the wound epidermis and inhibits the signaling cascades that initiate blastema formation. Fibronectin is an important substrate molecule for both migrating epidermal cells and fibroblasts of wounds. FN is strongly upregulated during blastema formation in axolotl limb regeneration [15]. A prerequisite for blastema formation and growth in urodele limb regeneration is the thickening of the initial wound epidermis to form the AEC. Fibronectin is an important component of the blastemal ECM and is highly expressed by the basal layer of the AEC starting within 24 hrs after amputation, as well as by blastema cells themselves [54,55]. The AEC appears to direct the migration of blastema cells to form the accumulation blastema beneath it. This was shown by experiments in which shifting the position of the AEC laterally caused a corresponding shift in blastema cell accumulation [56], and transplantation of an additional AEC to the base of the blastema resulted in supernumerary blastema formation [57]. The redirected accumulation of blastema cells in these experiments may be due to the migration of the cells on FN produced by the eccentric AEC. TGF-β1 is strongly up regulated during blastema formation in amputated axolotl limbs [52]. FN is a target gene of TGF-β1 that is highly expressed by basal cells of the wound epidermis during blastema formation [54]. Inhibition of TGF-β1 expression with SB-431542, reduces FN expression and results in failure of blastema formation [52], again suggesting that FN provided by the AEC provides directional guidance for blastema cells.

In the present study, we identified a canonical pathway in which TGF-β1 leads to the activation of SP1 through TGF-β receptors and SMAD3. Transforming growth factor-β1 is one of the major inducers of epithelial-mesenchymal transformation (EMT) via SMAD family member proteins (SMAD2, SMAD3 and SMAD4) [58,59]. The epidermal cells that establish the wound epidermis in regenerating urodeles limbs take on some of the characteristics of mesenchymal cells, shedding their specialized epithelial junctions and up regulating cytoskeletal components essential for migration. TGF-β1 binds Type I and type II receptor serine/threonine kinases. The receptor type II phosphorylates the receptor type I, which activate SMADs [31,32] and SMAD3 then leads to activation of SP1 which is capable of activating FN [30]. Interestingly, there is a non-canonical TGF-**β**1 pathway in which SMAD 3 can repress c-Myc through a novel repressive SMAD binding element within the TGF-**β **inhibitory element of the c-Myc promoter [60]. Wound epidermal cells migrating over the amputation surface do not divide [11]. In this context, SMAD3 could possibly inhibit the division of migrating epidermal cells via this pathway.

Figure 6 illustrates multiple pathways that lead to c-Myc activation from FN. The highlighted pathway is the longest canonical pathway and it involves the cell adhesion proteins talin, FAK1 (focal adhesion kinase1), c-Src, Paxillin, ILK (integrin-linked protein kinase) and components of the canonical Wnt signaling pathway (GSK3-β-glycogen synthase kinase-3 beta, β-catenin, and Tcf/Lef (T-cell-specific transcription factor/lymphoid enhancer-binding factor 1). Wnt signaling is known to control cell proliferation and cell fate determination. Members of the Wnt and BMP pathways have been shown to be required in vertebrates for normal limb development [61]. Canonical Wnt signaling is also known to keep stem cells in a self-renewing and undifferentiated state[62]. Loss- and gain-of-function experiments in axolotl, Xenopus, and zebrafish showed that Wnt signaling is required for limb and fin regeneration [61]. Another study in zebrafish and chick embryos has identified molecular interactions of Wnt2b with Tbx5 that are responsible for limb identity and outgrowth [63]. These findings indicate that Wnt signaling is probably required for the activation of c-Myc.

## Conclusions

In the present study we asked whether the use of proteins identified in a proteomic analysis of blastema formation in amputated axolotl hindlimbs could be used as bait to identify transcription factors and their downstream targets involved in blastema formation, and construct these into interactive protein networks and pathways. We identified multiple targets of c-Myc and SP1, and also several upstream molecules (TGF-β receptors, SMADs, and cell adhesion molecules) that lead to the activation of c-Myc and SP1. We conclude that this strategy can be successful, not only for transcription factors and their targets, but for other molecules as well that might be important to regeneration and/or wound repair.

The next step is to construct hypotheses that allow experimental testing of the roles of the molecules comprising the interactive protein pathways in regeneration-competent limbs. This will unite the question-driven approach used here with the hypothesis-driven approach. Both are equally important for analysis and synthesis of data derived from complex biological processes. We are currently testing one hypothesis about the role of the centrosomal protein Evi5 (ecotropic viral integration factor 5) and the pathways it forms with several other proteins regulating the cell cycle during blastema formation and growth [15].

Finally, by deriving proteomic data from a regeneration-deficient system such as the limbs of Xenopus froglets, and applying a bioinformatics/systems biology approach, we have the possibility of identifying a set of proteins, networks and pathways that can be compared to that of the regeneration-competent axolotl to reveal the basis for the difference between the two.

## Authors' contributions

DJ: conception of project, conception and conduct of experimental design, data analysis, writing and revision of manuscript, figure and table preparation; NR: source of proteomic data, manuscript critique and revision; DJM: writing of manuscript, manuscript critique and revision; FS: manuscript critique and revision; JAC: manuscript critique and revision; DLS: conception of project, data analysis, writing and revision of manuscript; MP: conception of project, bioinformatics tools, writing and revision of manuscript. All authors read and approved the final manuscript.

## Supplementary Material

Additional file 1 **Axolotl proteomics data with its respective human orthologs, gene symbol and fold changes at 1, 4, and 7 dpa**. FC_1 D refers to the fold change at 1 dpa with respect to control. FC_4 D and FC_7 D can be similarly interpreted. A negative value implies downregulation with respect to control and a positive fold change value implies upregulation. Click here for file

Additional file 2 **Biological process enrichment for the protein categories in **Figure 1. The DAVID tool was used to obtain the biological process enrichment for each category of proteins represented in Figure 1. The associated biological process terms, number of proteins (count), percentage, p-value, and genes obtained from the DAVID tool are mentioned for each group of proteins. Click here for file

Additional file 3 **The five most highly connected TFs for each up and downregulated group of proteins**. The table shows the GO processes, root nodes (the number of target proteins) and an overall p-value for each TF in the up or downregulated group. The numbers in parenthesis in the third column indicate the percentage of target proteins and a p-value for that particular GO term with respect to the target proteins of a given TF. The TFs are arranged in decreasing order of significance (higher p-value) within each group. Click here for file

Additional file 4 **The five most highly connected TFs with their target proteins**. The gene symbols (from additional file 1) are defined for the target proteins of each TF. Click here for file

Additional file 5 **Pathways for the target proteins of c-Myc and SP1 in each up and downregulated group of proteins**. The list of significant pathway names as obtained from MetaCore with their respective p-values for the target proteins of c-Myc and SP1 in each up and downregulated group of 1, 4 and 7 dpa. The pathways are arranged in decreasing order of significance within each group. Click here for file

Additional file 6 **Pathways for those target proteins not regulated by c-Myc and SP1 in each up and downregulated group of proteins**. The list of significant pathway names obtained from MetaCore, with their respective p-values. The pathways are arranged in decreasing order of significance within each group. Click here for file

Additional file 7 **Reference for the symbols used in the construction of networks**. Reference guide for various symbols used in the networks of Figures 3 to 7. Click here for file
