# Supplementary figures and images for: Network based transcription factor analysis of regenerating axolotl limbs
Source: BMC Bioinformatics. 2011 Mar 18;12:80. doi: 10.1186/1471-2105-12-80 (PMC3240668; doi:10.1186/1471-2105-12-80)

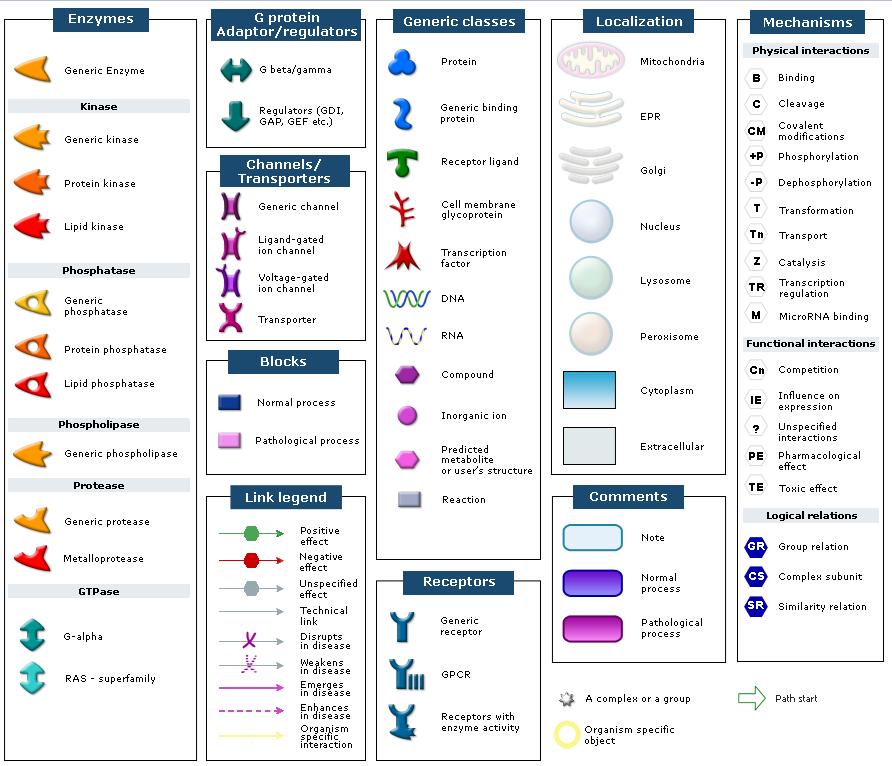

Supplement: Additional file 7 — Reference for the symbols used in the construction of networks. Reference guide for various symbols used in the networks of Figures 3 to 7. [file 1471-2105-12-80-S7.JPEG]
